# Some Observations on Puerperal Eclampsia

**Published:** 1894-01

**Authors:** Sam’l Cunningham

**Affiliations:** Elgin


For Texas Medical Journal.

### SOME OBSERVATIONS ON PUERPERAL ECLAMPSIA.

BY SAM'L CUNNINGHAM, M. D., ELGIN.

Read at the Austin District Medical Society, June 22, 1893.

IT WOULD seem like an unwarranted presumption, nor shall I attempt before this intelligent body to advance anything especially new upon this subject which has been so elaborately discussed by the numerous masters of the obstetric art. It is rather for the purpose of eliciting your discussions, thus availing myself of your views and experience that I have ventured to introduce this subject to your notice. Without further apologies I shall assume that there is no branch of obstetrics in which a thorough knowledge of what to do is more essential. Most certainly it is a condition accompanied by the most grave symptoms, and constitutes one of the most serious diseases with which the physician has to cope. Statistics have proven the disease of not very frequent occurrence, yet how often it seems the case when attending the parturient woman the gaze of the physician is horrified to witness these tonic convulsive epileptic-like seizures that so suddenly take hold of his patient, in the midst of which grave excitement many physicians almost lose their wits, realizing then that at most times assistance can not be procured, thus compelling the attendant to depend upon his own knowledge and resources. This fact so well recognized as true, should be sufficiently important to stimulate the doctor to get as thorough acquaintance with the subject as possible, together with all the means now adopted in the practice of midwifery and obstetrical surgery.

In the majority of these eclampsiæ there are premonitory symptoms which announce to the wide awake attendant the threatening convulsion. Prominent among these symptoms which should serve to remind the attendant of danger, are these: The woman complains of headache, dizziness; she shows loss of memory, becomes gloomy and despondent, often assumes a peaceful and quiet slumber, a condition described by Prof. Lusk, in his text book on midwifery, as "a deceitful truce," or a "state in which the woman ceases to complain." After this state of calmness, which usually lasts from one to five minutes, she suddenly opens her eyes which appear to be fixed upon some object. Next her face is adorned with a silly smiling aspect, the pupils can be seen rapidly dilating. These are a few of the symptoms which my experience, though comparatively limited, have invariably verified as being precedents of the first convulsions.

Upon reviewing the history of eclampsia and the views of prominent observers since earliest obstetrical records, it is clearly to be seen that the disease is not dependent upon one and the same cause; hence, the necessity of cautiously adopting such treatment as is peculiarly and most intelligently indicated in the management of each particular case and its symptoms.

Since albuminuria has been so largely discussed and has proven so important a factor in accounting for the occurrence of these eclampsiæ, and since the œdema and anasarca in the majority of cases present such bold intimations of the possible advent of this disease, it should be the duty of every practitioner, in order to defend against this formidable disease, the pregnant women committed to his charge, to exercise diligence, and carefully watch after her, by frequent examinations of the urine.

The presence of albumin, especially if to any great extent, demands the strictest prophylactic measures, and every precaution should be taken to lessen this abnormality by employing such treatment as will induce the skin and bowel to aid the kidneys, the woman being supported by tonics, and strict dietetic measures should be enjoined upon her.

The frequency of eclampsia is estimated by Prof. Lusk to be about one in every five hundred pregnancies. My own experience, however, is to have seen the disease more often than is indicated in this report, as in my individual practice I have officiated as accoucheur, up to the present time, in one thousand and seventy-two cases of labor at full term, and have met with six cases of eclampsia. Besides this, I have seen two other cases in consultation, which makes an experience of eight cases that have come under my immediate observation.

With these impe:fect preliminary remarks, especially since I did not start out to enlighten you by presenting to you a beautiful etiology of this disease, I shall now proceed to describe the eight cases to which allusion has been made.

They were all primiparæ. The presentations were all vertex. The sex of the child, in every instance, was male. Six of these cases were of the ante-partum and two of the post-partum variety. Of these eight cases, it affords me no small degree of pleasure to report seven recoveries, several of whom still survive, and within the bounds of my territory, continue to propagate their species; and I am honored with a call to officiate every eighteen months or so. The first case (and, too, the one who died), of whom I shall speak, was Mrs. J. T. L., age 20, rather stout, was taken in labor on the 13th day of November, 1884. I was called in great haste on the 15th, two days later than beginning of labor. The messenger apprised me of the fact that she was having convulsions, and that the child was yet unborn, and of the attendant's desire that I should come prepared for the most extreme emergency. The distance was about eight miles, and over a rough road. Upon arrival I found her in the most violent convulsions. The attendant, Dr. Bragg, who lives in that community, estimated the number of attacks to have been about forty prior to delivery, which we accomplished by the use of the forceps, after placing her under the influence of chloroform. In this instance there was complete uterine inertia, the os only partially dilated, though not rigid. The point of greatest difficulty was the high position of the head, it being about the superior strait. These were serious hindrances which greatly complicated the condition, and made it exceedingly difficult to apply the forceps, also required greater traction to effect delivery than would have been otherwise required, had circumstances been more favorable. The child had evidently been dead for some days, as it was almost black, and the cuticle became denuded from the shoulders and hips, on delivery. She lived three days after delivery, and continued to have an occasional convulsion up to her death, in spite of the effect of chloral, and bro. pot., and digitalis, which were administered in full doses. After delivery, the entire uterine cavity was thoroughly irrigated by hot antisepsic fluid, but she died with marked symptoms of septicæmia and meningitis.

Case No. 2, to which I will introduce you, is Mrs. S. H. M., whose age was 28, quite a stout, fleshy and beautiful woman. She began labor on the 5th day of August, 1887, and after an exhaustive, tedious struggle of 24 hours, gave birth to a well-developed boy of 9 pounds on August 6th, at 5 p. m. Immediately on birth of the child, before delivery of the placenta, she was seized in a most violent convulsion. In the midst of the great excitement chloroform was given, and as the convulsion was abating, a good dose of morphine and atropia was given hypodermically. Placenta was easily expressed. Watching her closely for the next twenty-four hours, as she would become restless, and pulse inclined to beat too fast, I, for the first time in my experience, employed the much lauded veratrum, giving it only when the circulation was accelerated, in ten-drop doses, hypodermatically, and at such intervals as her symptoms would indicate.

In honor to those whose experiences are so favorable as to have so much eulogized this drug, I feel inclined to express myself indebted for the happy influence it undoubtedly wielded upon this patient. She had no return of the convulsions, and made as quick recovery as any other case that I have ever seen.

Case No. 3, Mrs. B. P., age 16, rather a delicate girl; gestation having gone hard with her; very anasarcous, and pale. On the 26th day of March, at 9 o'clock p. m., she was on the first warning of labor, seized with a most violent convulsion. Living only a few blocks from her, and not having gone to bed, I was soon in attendance. When I got to her bedside the attack had about subsided, but she could not be aroused. A second convulsion was soon on her, while I was preparing my hypodermic syringe for use. I at once injected the dose, and gave chloroform. Digital examination revealed an undilated, rigid os and vagina. The nervous and circulatory system were maintained in a state of quietude, as symptoms indicated, by a hypodermic of morphine, ergot and veratrum. The bowels being constipated, she was given a large dose of castor oil, turpentine and calomel, which acted well. She soon regained her consciousness, and with only the above described treatment her labor assumed a normal course, and at 11 o'clock p. m. of March 29th, by the natural forces, she gave birth to a well-developed boy which lived and did well. Her labor lasted three days.

Case No. 4, Mrs. R. L. B., age 19. She gave the history of having been very stout before marriage, when she began to have great trouble with her menstrual periods, making her sick enough to go to bed for several days, and lost much flesh. After becoming pregnant, her health did not improve much, as morning sickness was a serious complication of her condition, and gestation had gone hard with her all along. Was first called to her on the morning of the 15th day of September, 1890. Digital examination revealed an undilated and rigid cervix, all symptoms pointing to a tedious and hard time. I left her, instructing her husband to send back for me when the pains began to increase. Was called back to her that night, having to get up from bed and go a distance of three miles to her. Digital examination, at this time, revealed but little change. A good hypodermic of morphine afforded her some ease from the spurious pains, and she slept some. Shortly the pains seem to have begun in good earnest, and the os began to dilate. I felt hopeful of seeing a favorable termination of the labor in a few hours, and I could be released, but to my sorrow, after a few hours' anxious waiting, she suddenly exclaimed, "What's the matter with me? I can't see; light a lamp; I'm blind; I'm going to die."

Recognizing these and other symptoms, as grave concomitants of an impending convulsion, I proceeded to administer chloroform, and as quietly and rapidly as possible applied the forceps and was successful in accomplishing delivery in but a short time. After a few minutes delay the placenta was carefully and easily expressed, and when I began to indulge in the pride of having in this instance protected my patient from the ordeal I had so much dreaded, she was seized in a hard convulsion. Chloroform was immediately resumed, and as the attack was subsiding, a hypodermic of morphia and atropia was given. A few ten drop doses of n. t. veratrun combined with twenty drops f. e. ergot, and an eighth gr. morph. was given at intervals as indicated to promote quietude of nervous and circulating system. Oil and turpentine given as a usual custom. She had but the one convulsion mentioned, and without having any fever went on to a rapid and good recovery. The child is still living, and a stout boy.

Case No. 5, Mrs. C. T. O., age 34. Her physical conformation was tall and slender, married at the age of 33 years. Had spent ten years prior to marriage, in teaching school, and was of an exceedingly high nervous temperament. She began labor on the morning of the 15th day of November, 1890. Was called to see her same day at 9 p. m. Found her progressing nicely in the first stage of labor; the conditions then appearing favorable for an early and safe termination. Having noticed some anxiety on the part of her husband, an Irishman, I at once apprised him of my understanding of his wife's condition, which explanation occasioned him much vexation and displeasure, and he retorted by saying: "Doctor, you are mistaken. It is not time for my wife to be sick, for we have only been married nine months to-day." I explained to him as best I could the laws regulating gestation, but to avail nothing, as he as well as she was inexorable in the belief that I should institute some treatment that would arrest the labor, "at least for a few days." The unpleasantness of the surroundings became so intense that I could not resist tendering my resignation, and as nicely as I could insisted that they call some one else who might possibly serve them better than I was capable of doing. But they were complimentary enough to express their preference for my remaining, "if it did have to come."

But back to the case, and apoligizing for having trespassed upon your patience in such a discussion of the subject. After the lapse of a few hours, she was in the hardest labor pains. Grave symptoms began to present; to be brief, she was failing; physical exhaustion was a prominent feature. I told her husband what I feared would be the result of further delaying the use of the forceps, but his prejudice for such procedure was so great that I was denied the exercise of my conservatism until she was in convulsions, which occurred at 5 o'clock a. m., being the 16th day of November, 1890. Chloroform anesthesia first obtained, she was carefully delivered by first using the forceps, of a large, well developed boy, which is still growing finely. She had no return of convulsions, sustaining no laceration, had no fever, and with no other treatment than a dose of morph. and artopia subcutaneously injected after delivery, a dose of castor oil and turpentine, and a few doses of quinine made an early and good recovery. Of the remaining three cases I have notes, but upon reviewing them in preparing this paper, find such a general similarity of features in them all to each other, I will not burden your patience by detailing them singly, but will only speak of them in a general way.

In each instance the convulsions were prior to the delivery. No return of convulsious after delivery in any of them. Chloroform anesthesia, careful adjustment of forceps, slowly and cautiously bringing the head of the child well down on the perineum, at which time the instruments were removed, the labor conducted with my hand. Towels wrung from hot water were kept constantly applied to the perinæum which favored relaxation, till expulsion of the head, were about the treatment in these trying ordeals, all of which terminated safely to mothers and babies. In all these eight cases herein so imperfectly described, excepting the first case, I have made analysis of the urine, my observations being to find in all cases examined the presence of albumen in considerable quantity. Which fact impresses me, and leads me to better appreciate the researches of those who have in statistics so frequently shown the association of albuminuria with "puerperal eclampsia." Since in these eclampsiæ there is such abundant reason for supposing the presence of renal insufficiency I endeavor to restore the kidneys to their normal functions, as well as the introduction of such agents best calculated to lower arterial tension, thereby diminishing the great excitement of the vaso-motor centers are among the first most prominent indications of treatment. Venesection has many advocates in bringing about the better state of affairs at this particular time. While I have never had any experience in bleeding, am free to confess a profound regard for those who have so intelligently based their opinions, especially in some forms of the disease. Since these convulsions have a tendency to continue as long as the labor lasts, and since the frequency of their occurrence so greatly enhances the danger to the woman, every obsterical resource consistent with safety, in my humble opinion is speedily demanded of the attendant to hasten the delivery.

As a rule, I regard a hypodermic of morph. and atropia after delivery indespensable, especially where the conditions are so serious as to have demanded the use of the forceps. Then the nervous system, if you please, should be kept in a state of quietude as long as symptoms indicate a necessity, by giving chloral and bromide of potassium.

Norwood's tinct. of veratrum viridi should be cautiously given hypodermically when the pulse rate is greatly accelerated.

